# Attenuation of 6‑OHDA-Induced Neurotoxicity by 1,2,3-Triazole-Based Sulfonamides through SIRT1 Activity

**DOI:** 10.1021/acsomega.5c03278

**Published:** 2025-06-27

**Authors:** Papitcha Jongwachirachai, Ratchanok Pingaew, Veda Prachayasittikul, Waralee Ruankham, Setthawut Apiraksattayakul, Wilasinee Suwanjang, Tanawut Tantimongcolwat, Virapong Prachayasittikul, Supaluk Prachayasittikul, Kamonrat Phopin

**Affiliations:** † Center for Research Innovation and Biomedical Informatics, Faculty of Medical Technology, 549272 Mahidol University, Bangkok 10700, Thailand; ‡ Department of Chemistry, Faculty of Science, 37692 Srinakharinwirot University, Bangkok 10110, Thailand; § Department of Clinical Chemistry, Faculty of Medical Technology, 26685 Mahidol University, Bangkok 10700, Thailand; ∥ Department of Clinical Microbiology and Applied Technology, Faculty of Medical Technology, 203384 Mahidol University, Bangkok 10700, Thailand

## Abstract

Due to the irreversible destructive nature of Parkinson’s disease (PD), the development of neuroprotective agents is crucially needed as its current medications are only symptomatic treatments. 1,2,3-Triazole is an attractive scaffold for the discovery of novel therapeutic agents due to its versatile biological activities. This study aimed to investigate the neuroprotective effect of 1,2,3-triazole-based sulfonamides against 6-hydroxydopamine (6-OHDA)-induced neuronal SH-SY5Y cells. The effects of triazole derivatives on cell viability, intracellular reactive oxygen species (ROS) production, lactate dehydrogenase (LDH) leakage, and sirtuin 1 (SIRT1) activity were evaluated in the 6-OHDA-induced cells. The results indicated that six compounds (**2a** – **5a** and **3b** – **4b**) exhibited promising neuroprotective potentials due to their abilities to decrease intracellular ROS production and LDH leakage, along with increasing SIRT1 activity. Molecular docking was also conducted to reveal possible binding modes and interactions against the SIRT1 target through the key interacting residues of ILE223, LEU215, and PRO212. *In silico* pharmacokinetic prediction indicated that these compounds are drug-like molecules, possibly further developed as oral neuroprotective drugs. A set of possible PD-related targets of these six compounds is also highlighted for further investigations. Cytotoxicity assays in normal lung MRC-5 fibroblasts showed relatively high IC_50_ values, indicating low toxicity and favorable safety profiles. In summary, these six 1,2,3-triazole-based sulfonamides might potentially be neuroprotective agents for further PD therapeutic development.

## Graphical abstract

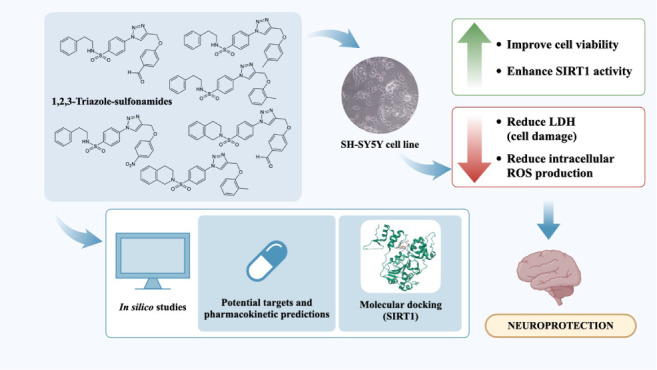

## Introduction

Aging population is increasing worldwide. According to the World Health Organization (WHO), the number of population aged equal to or over 65 years will increase to reach approximately 16% of the total population in 2050. This aging situation is also linked to an increased prevalence of chronic and degenerative diseases, including Parkinson’s disease (PD).^1^ PD is the second most common brain disorder found in the elderly, with the number of diagnosed patients having increased significantly over the last decades.^2,3^ The disease is manipulated by the progressive loss or dysfunction of the neurons located in the substantia nigra pars compacta, leading to the decline production of the dopamine, which is the major neurotransmitter of motor function.^4^ This leads to several clinical complications, including nonmotor (i.e., cognitive impairment) and motor (i.e., resting tremors, rigidity, postural instability, and bradykinesia) symptoms.^5,6^ The precise mechanism underlying the degeneration of dopaminergic neurons remains unclear. However, several studies suggested that oxidative stress could be one of the key players.^7,8^ Reactive oxygen species (ROS) are reactive derivatives of oxygen mainly produced intracellularly by the mitochondria. Previous evidence indicated the elevations in oxidized forms of lipid,^9^ protein,^10^ and DNA (i.e., protein deglycase DJ-1 ^11^ and 8-oxoguanine (8-oxoG))^10,12^ in individuals with PD, which suggested the key roles of oxidative stress in the disease.^11^

While the progressive loss of dopaminergic neuronal cells is an irreversible process, current drugs are only symptomatic therapeutics that are unable to slow down the progression of the disease.^4,13^ Most of the drugs aim to increase the level or action of dopamine, such as levodopa,^13^ monoamine oxidase (MAO) inhibitors, and dopamine antagonists.^14^ Due to the irreversible and progressive nature of PD, prevention and delaying the progression at the early stage of disease is essential. Accordingly, the development of novel potent neuroprotective agents is of great interest. 1,2,3-Triazole is a heterocyclic nitrogen-containing pharmacophore popularly used as a template for drug development.^15^ The 1,2,3-triazole derivatives exhibit various pharmacological effects related to neuroprotection, such as anti-inflammatory,^16^ antiapoptotic,^17–19^ α-synuclein inhibitory,^18^ and amyloid beta (Aβ)-aggregation modulatory^19^ activities. For example, triazole-pyrimidine hybrids have been reported to modulate neuroinflammation and promote neuroprotection in the microglial HMC-3 cells.^17^ In the PD model, triazoles were reported to act as potential α-synuclein inhibitors, showing binding affinity toward α-synuclein as well as the ability to inhibit its aggregation.^18^ This suggests that 1,2,3-triazole is an attractive scaffold for the discovery of novel neuroprotective agents.

Drug development process is widely recognized for its costly, lengthy, complex, and high-rate failure nature.^20^ Currently, computational approaches are commonly used tools to facilitate successful drug discovery and development.^21^ Molecular docking is a well-recognized method to effectively reveal favorable binding modes and key interactions of compounds with their biological targets.^22^ This could facilitate the effective design or structural optimization of related compounds to obtain new derivatives with improved properties. A major proportion of drugs failed during the clinical phases, often due to their considerable toxicities or unpreferable efficacies.^23^ Accordingly, the *in silico* predictions of drug-likeness or pharmacokinetic profiles at an early stage of development are currently encouraged to prioritize compounds with potential for success.^24^ A series of 1,2,3-triazole-based sulfonamides was previously reported as aromatase inhibitors^25^ and anticancer agents^26^ by our research group. However, the neuroprotective effects of these synthetic compounds have not yet been elucidated. In this study, these triazole derivatives were investigated for their protective effects against 6-hydroxydopamine (6-OHDA)-induced neurotoxicity in the neuroblastoma SH-SY5Y cell line, as well as their cytotoxicities in normal lung MRC-5 fibroblasts. *In silico* studies (i.e., molecular docking, pharmacokinetic prediction, and potential target prediction) were also performed to gain insightful knowledge toward further design and development for PD management.

## Materials and Methods

### Reagents and Chemicals

Dulbecco’s modified Eagle medium (DMEM), fetal bovine serum (FBS), penicillin-streptomycin solution, 0.25% trypsin-EDTA solution, and 0.4% trypan blue solution were acquired from Gibco BRL (Gaithersburg, MD, USA). Sodium bicarbonate (NaHCO_3_), sodium chloride (NaCl), potassium chloride (KCl), sodium phosphate dibasic (Na_2_HPO_4_), monopotassium phosphate (KH_2_PO_4_), 3-(4,5-dimethylthiazol-2-yl)-2,5-diphenyltetrazolium bromide (MTT), dimethyl sulfoxide (DMSO), 2′,7′-dichlorodihydrofluorescin diacetate (H_2_DCFDA), rhodamine 123, LDH assay kit (MAK066), SIRT1 activity kit (CS1040), protease inhibitor, 1X RIPA buffer, and 6-OHDA were purchased from Sigma-Aldrich (St. Louis, MO, USA). Bradford protein assay reagent was obtained from Bio-Rad Laboratories (Hercules, CA, USA).

### Synthesis of 1,2,3-Triazole-Based Sulfonamide Derivatives

1,2,3-Triazole-based sulfonamide derivatives (Figure 1) were synthesized by our group^25,26^ using the copper­(I)-catalyzed azide-alkyne cycloaddition (CuAAC) as a key step to construct the desired 1,2,3-triazole ring. Then, the chemical structures of the synthesized compounds were confirmed by ^1^H and ^13^C NMR, IR, and HRMS data.

**Figure 1:**
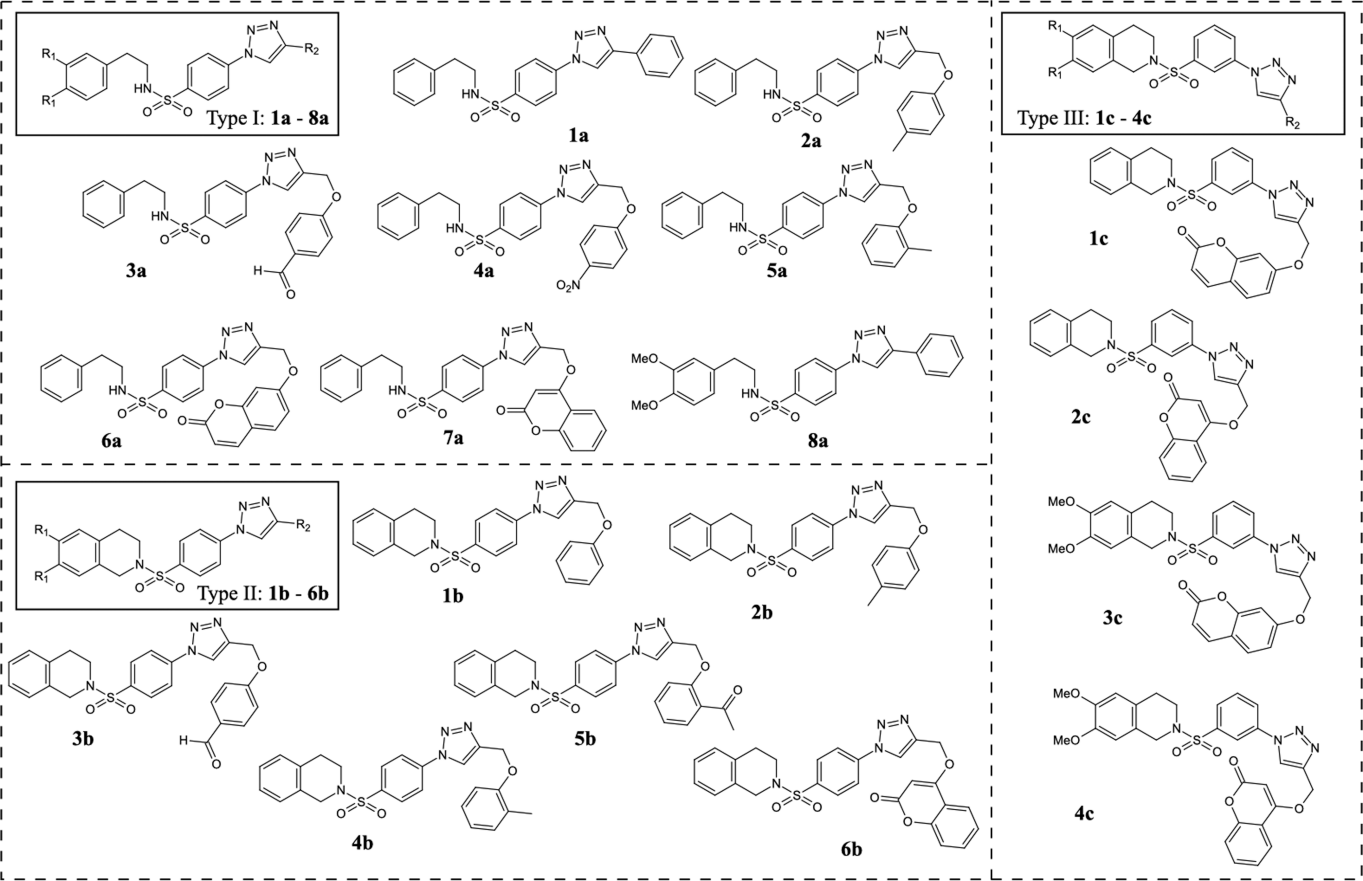
Chemical structures of 1,2,3-triazole-based sulfonamides, categorized into 3 groups as follows: type I (**1a** – **8a**), type II (**1b** – **6b)**, and type III (**1c** – **4c**).

### Culture of MRC-5 and SH-SY5Y Cells

Normal embryonic lung MRC-5 cells and neuroblastoma SH-SY5Y cells from the American Type Culture Collection (Manassas, VA, USA) were maintained in DMEM with 10% heat-inactivated FBS and 1% penicillin-streptomycin at 37 °C in humidified air containing 5% carbon dioxide.

### Cytotoxicity of 1,2,3-Triazole-Based Sulfonamide Derivatives on MRC-5 Cell Line

Cytotoxicity of 1,2,3-triazole-based sulfonamides was evaluated by using a normal embryonic lung (MRC-5) cell line. The MRC-5 cells were cultured in 96-well microtiter plates (Corning Inc., NY, USA) at 5,000–20,000 cells/well and incubated for 24 h at 37 °C in humidified air containing 5% carbon dioxide. Subsequently, various concentrations of 1,2,3-triazole derivatives, doxorubicin (used as a positive control), and DMSO (used as a negative control) were added and incubated for an additional 48 h.^27^ MTT reagent (0.5 mg/mL in medium) was added for cell staining and incubated for 4 h. Then, the remaining formazan was dissolved in DMSO by sonication. The absorbance of the test wavelength of 550 nm and the reference wavelength of 650 nm was analyzed with a microplate reader (Molecular Devices, CA, USA). IC_50_ of each compound was determined as the concentration of the compound that inhibits cell growth by 50%. Compounds with an IC_50_ greater than 50 μM were considered noncytotoxic.

### Determination of SH-SY5Y Cell Viability by MTT Assay

Prior to investigations, 1.0 × 10^5^ SH-SY5Y neuronal cells/mL were seeded into 96-well plates for 24 h. To determine the optimal concentration of 6-OHDA for inducing neurotoxicity, cells were seeded into 96-well plates for 24 h and treated with various concentrations of 6-OHDA ranging from 25 to 125 μM for another 24 h.^28^ For the evaluation of the cytotoxicity of 1,2,3-triazole-based sulfonamide derivatives on SH-SY5Y neuronal cells, the cells were treated with various concentrations (0.1–10 μM) of 1,2,3-triazole-based sulfonamide derivatives for 24 h.^29^ To assess the neuroprotective activity of 1,2,3-triazole-based sulfonamide derivatives, the cells were pretreated with various concentrations (0.1–10 μM) of 1,2,3-triazole-based sulfonamide derivatives for 3 h before being treated with 50 μM of 6-OHDA for another 24 h. Cell viability was assessed by incubating with 5 mg/mL of MTT reagent for 3 h at 37 °C, followed by absorbance measurement at 570 nm using a microplate reader (Thermo Fisher Scientific Inc., MA, USA). The viability of SH-SY5Y cells at each concentration was analyzed as a percentage relative to the control group.

### Morphological Assessment by Light Microscope

SH-SY5Y cells at a density of 1.0 × 10^5^ cells/mL were cultured in 6-well plates and treated as mentioned previously at 37 °C in humidified air with 5% carbon dioxide. The morphology of neuronal cells under different conditions was observed under an inverted light microscope (Olympus Corporation, Tokyo, Japan) at 40× magnification. Multiple images were taken for an individual treatment using a digital camera.^30^

### Measurement of ROS Production

ROS production in SH-SY5Y neuronal cells was investigated using H_2_DCFDA. 1.0 × 10^5^ cells/mL of SH-SY5Y neuronal cells were seeded into 96-well plates for 24 h, and 5 μM of selected 1,2,3-triazole-based sulfonamide derivatives were pretreated for 3 h. Then, neurotoxicity of the cells were induced with 50 μM of 6-OHDA for another 24 h. The neuronal cells with 10 μM H_2_DCFDA were incubated for 30 min at 37 °C in the dark. The emission and excitation wavelengths of 528 nm and 485 nm, respectively, were measured by using a microplate reader. The measured ROS production was calculated as a percentage relative to the control group.^31^

### Evaluation of Cellular Leakage of LDH Enzyme

Leakage of LDH, an enzyme commonly used as an assessment factor to evaluate cellular damage, was measured to determine the protective ability of the compounds against 6-OHDA-induced neuronal damage. 1.0 × 10^5^ cells/mL of SH-SY5Y neuronal cells were seeded into 6-well plates for 24 h. The cells were pretreated with 5 μM of 1,2,3-triazole-based sulfonamide derivatives for 3 h before being treated with 50 μM of 6-OHDA for 24 h. The cell culture medium of SH-SY5Y cells was collected, and LDH leakage was evaluated using the LDH assay kit. In the presence of LDH, lactate is converted into pyruvate, resulting in an elevated level of NADH production, which is determined via the change in absorbance at 450 nm. LDH activity was calculated according to the manufacturer’s instructions and shown as a percentage of the control group.^32^

### Determination of SIRT1 Deacetylase Activity

SH-SY5Y neuronal cells at 1.0 × 10^5^ cells/mL were seeded into 6-well plates for 24 h. Then, the cells were pretreated with 5 μM 1,2,3-triazole-based sulfonamide derivatives for 3 h before neurotoxicity was induced with 50 μM 6-OHDA for another 24 h. The cell culture medium was rinsed off with cold 1X PBS. The cells were incubated at 4 °C with 1X RIPA buffer containing a protease inhibitor for 20 min before being harvested using a cell scraper, followed by centrifugation at 12,000 rpm for 20 min. Protein concentrations were measured using the Bradford protein assay, and SIRT1 activity in the cells was determined following the manufacturer’s instructions. The increase in SIRT1 enzyme activity was measured by fluorescence intensity at an excitation wavelength of 340 nm and an emission wavelength of 445 nm. SIRT1 enzyme activity was statistically analyzed as a percentage relative to the control group.^33^

### *In Silico* Pharmacokinetic Properties Prediction

Physicochemical properties (i.e., molecular weight (MW), number of rotatable bonds, topological polar surface area (TPSA), etc.), and pharmacokinetic properties (i.e., absorption, distribution, metabolism, and excretion) of the studied triazoles were predicted using web-based tools SwissADME (http://www.swissadme.ch) and pkCSM (https://biosig.lab.uq.edu.au/pkcsm/). The chemical structures of the compounds in SMILES format were used as inputs for the predictions. Drug-likeness of the compounds was determined using (i) Lipinski’s rule of five and (ii) Veber’s rule. Drug-like compounds should comply with at least three criteria of Lipinski’s rule regarding size (MW ≤ 500 g/mol), lipophilicity (−2 ≤ LogP ≤ 5), and number of hydrogen bond acceptors (N or O ≤ 10) and donors (NH or OH ≤ 5),^34^ while the Veber’s rule includes additional properties for consideration, such as TPSA ≤ 140 Å^2^ and number of rotatable bonds ≤ 10.^35^ Additionally, toxicity profiles of the compounds were predicted using ProTox-II online software (http://tox.charite.de/protox_II).

### Molecular Docking

Molecular docking was performed to predict possible binding modalities of the studied compounds against the activator binding site of the target enzyme, SIRT1 deacetylase. The crystal structure of the target protein SIRT1 in complex with resveratrol and an AMC-containing peptide (PDB ID: 5BTR) was obtained from the protein data bank. Prior to the docking process, the cocrystallized ligands were removed from the protein structure, and only chain A of the protein structure was selected for the docking process. Hydrogen atoms were added to the protein structure, and the Kollman charge was calculated. The ligands (i.e., 1,2,3-triazole-based sulfonamide derivatives and the cocrystallized resveratrol) were prepared by adding hydrogen atoms and assigning the Gasteiger atomic partial charges. Docking simulations were conducted using AutoDockTools version 4.2.6.^36^ A grid box size of 60 × 60 × 60 points was allocated to cover the binding site of resveratrol, which was located at the catalytic and extended N-terminal domain of SIRT1. Rotational bonds of the ligands were set as flexible, while the protein structure was set as rigid. The docking was simulated for 100 runs of the Lamarckian genetic algorithm,^37^ with other parameters set as defaults. Prior to the simulations using the studied compounds, the redocking of the cocrystallized resveratrol was performed to validate the docking protocol. The root-mean-square deviation (RMSD) of the predicted binding pose of resveratrol was calculated. The docking results were ranked according to the binding energy score (ΔG), and the conformations with the lowest binding energy were selected for further analysis. Ligand-protein binding interactions in 2D and 3D diagrams were visualized using Discovery Studio Visualizer version 21 (BIOVIA, Dassault Systèmes).

### Target Prediction

Chemical structures in SMILES format were used to predict possible targets of compounds using three online web-based tools (i.e., SwissTargetPrediction (http://www.swisstargetprediction.ch/),^38^ SuperPred (https://prediction.charite.de/index.php?site=chemdoodlesearch),^39^ and Similarity Ensemble Approach (SEA) database (https://sea.bkslab.org/).^40^ For target proteins related to PD, a list of PD-related targets was created by searching across three databases (i.e., GeneCards (https://www.genecards.org/),^41^ DisGeNET (https://www.disgenet.org/),^42^ and Comparative Toxicogenomics Database (CTD) (http://ctdbase.org/)^43^ using the term “Parkinson’s disease”. To identify the proteins highly associated with PD, *Homo sapien*-specific genes with high gene-disease associated score (gda > 0.2) in DisGeNET was selected to fabricate the database.^44^ Then, the proteins related to the listed genes were obtained from UniProtKB (https://www.uniprot.org/help/uniprotkb).

Visualization of common targets between compounds and PD was illustrated using a Venn diagram (https://bioinfogp.cnb.csic.es/tools/venny/index.html)^45^ and a compound-target network (Cytoscape 3.10.1). The protein–protein interaction (PPI) networks were obtained from STRING (https://string-db.org/) database and illustrated using Cytoscape 3.10.1 software. Additionally, network topology parameters (i.e., degree, betweenness, and closeness centralities) were determined using a Network Analyzer. Degree centrality was determined through the number of edges with a direct link to the nodes of interest.^46^ A node with a higher degree centrality has a higher influence on other nodes and is probably an essential protein in the networks.^47^ Betweenness centrality measures the shortest distance that the node lies between the paths of two distinguished nodes.^48^ Closeness centrality was determined by the average distance of the shortest path for the node to reach all the other nodes in the network.^49^ Nodes with higher values in the betweenness and closeness centralities were considered to have greater influence in the networks.^39^

### Statistical Analysis

All data were expressed as mean ± standard error of the mean (SEM) values of five independent experiments. Statistical differences were determined by one-way analysis of variance (one-way ANOVA) using GraphPad Prism 8 scientific software (GraphPad Software Inc., CA, USA). Probability value (*p*) < 0.05 was statistically significant.

## Results

### Synthesis of 1,2,3-Triazole-Based Sulfonamide Derivatives

A set of eighteen 1,2,3-triazole-based sulfonamides (**1a** – **8a**, **1b** – **6b**, and **1c** – **4c**) was synthesized and characterized as previously reported by our group.^25,26^ The chemical structures of the synthesized compounds are listed in Figure 1.

### Cytotoxicity of 1,2,3-Triazole-Based Sulfonamide Derivatives Against Embryonic Lung MRC-5 Cell Line

Cytotoxicity of the studied triazoles on the MRC-5 cell line was assessed using the MTT assay (Table 1). All compounds showed lower cytotoxicity (IC_50_ of 14.38–111.47 μM) compared to the reference drug, doxorubicin (IC_50_ = 2.57 ± 0.36 μM).

**Table 1:** Cytotoxic Activity of Triazole-Based Sulfonamides (**1a** – **8a**, **1b** – **6b**, and **1c**– **4c**) Against Normal Embryonic Lung MRC-5 Cell Line

Triazole-based sulfonamides	IC_50_ (μM)
**1a**	102.68 ± 1.31
**2a**	89.40 ± 2.95
**3a**	>108.10
**4a**	80.39 ± 3.79
**5a**	>111.47
**6a**	55.34 ± 1.45
**7a**	>99.49
**8a**	78.81 ± 8.11
**1b**	59.03 ± 3.69
**2b**	70.42 ± 6.80
**3b**	>105.37
**4b**	19.04 ± 0.89
**5b**	15.41 ± 1.10
**6b**	>97.17
**1c**	14.38 ± 0.34
**2c**	>97.17
**3c**	>87.02
**4c**	>87.02
Doxorubicin^a^	2.57 ± 0.36

a Doxorubicin was used as a reference drug.

### Effect of 6-OHDA on Cell Viability of Neuroblastoma SH-SY5Y Cells

6-OHDA, a well-known catecholamine analogue as a neurotoxicant against the dopaminergic neurons, is widely used to mimic the PD condition in several studies. The neuronal toxicity of 6-OHDA has been reported to occur via the modulation of many oxidative stress-related pathways.^50^ Previous studies revealed that 6-OHDA significantly increased the level of apoptosis via an induction of lipid peroxidation in the PC-12 cell line^51^ as well as escalated the ROS production in the SH-SY5Y cell line.^52^ Furthermore, the 6-OHDA was reported to induce neuroinflammation and oxidative stress, leading to a decline of cognitive and motor functions.^53^ Cytotoxicity of 6-OHDA against SH-SY5Y cells was determined via the MTT assay after the cells were treated with various concentrations (25–125 μM) of 6-OHDA for 24 h. The results showed a significant decrease in viability of the cells treated with 50 to 125 μM 6-OHDA compared to the control (*p* ≤ 0.05). Moreover, the reduction of cell viability in a dose-dependent manner (89.99 ± 2.810%, 78.50 ± 2.734%, 61.65 ± 2.435%, 56.19 ± 2.368%, and 54.39 ± 3.063% at 25–125 μM 6-OHDA, respectively) was observed, as shown in Figure 2. Thus, a concentration of 6-OHDA at 50 μM was selected for further experiments to determine the neuroprotective properties of compounds on 6-OHDA-induced SH-SY5Y cell death.

**Figure 2:**
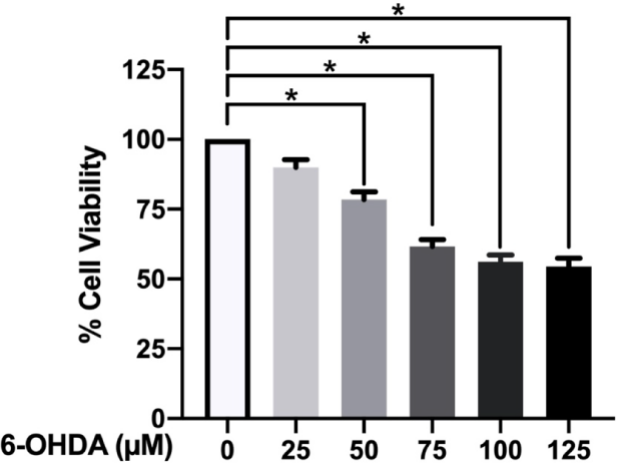
Cytotoxicity of 6-OHDA at various concentrations (25–125 μM) against SH-SY5Y neuronal cells after 24 h of treatment at 37 °C, evaluated by MTT assay (*N* = 5). Data are presented as mean ± SEM of the percentage relative to the control group. The statistical analysis was performed using one-way ANOVA. *p ≤ 0.05 compared to the untreated control group.

### Effect of 1,2,3-Triazole-Based Sulfonamide Derivatives on Cell Viability of 6-OHDA-Induced SH-SY5Y Cells

The neuroprotective effect of 1,2,3-triazole-based sulfonamides (**1a** – **8a**, **1b** – **6b**, and **1c** – **4c**) was determined using the MTT assay. As shown in Figure 3a, cells pretreated with the tested compounds at 0.1 to 10 μM showed no significant decrease in cell viability compared to the control group (*p* ≤ 0.05). Pretreatment with compounds **2a** – **5a** and **3b** – **4b** at 1 μM (90.11 ± 5.567%, 85.36 ± 4.615%, 88.68 ± 3.959%, 91.89 ± 9.100%, 89.37 ± 4.597%, and 88.69 ± 4.465%, respectively) and 5 μM (89.25 ± 3.236%, 87.69 ± 4.276%, 85.48 ± 4.470%, 90.88 ± 6.158%, 90.41 ± 7.193%, and 84.03 ± 1.563%, respectively) significantly increased the cell viability of the 6-OHDA-induced cell death (*p* ≤ 0.05). Resveratrol, a well-known as a neuroprotective agent, was used as a reference compound. The results indicated that pretreatment with resveratrol at 0.1–10 μM increased the cell viability of 6-OHDA-induced cells (90.01 ± 4.738%, 88.04 ± 6.146%, 92.06 ± 9.069%, and 90.47 ± 9.763%, respectively) compared to the 6-OHDA group (*p* ≤ 0.05). Additionally, morphological observations revealed that 6-OHDA-induced SH-SY5Y cells showed a round and detached morphology compared to the control group, which showed the normal spindle-shaped neuronal cells. However, pretreatment of the selected derivatives conserved the cellular morphology and exhibited fewer round and detached cells compared to the 6-OHDA-induced SH-SY5Y cells, which supported the results from the cell viability assay (Figure 3b). Thus, a set of compounds (**2a** – **5a** and **3b** – **4b**) at 5 μM was selected for further investigations.

**Figure 3:**
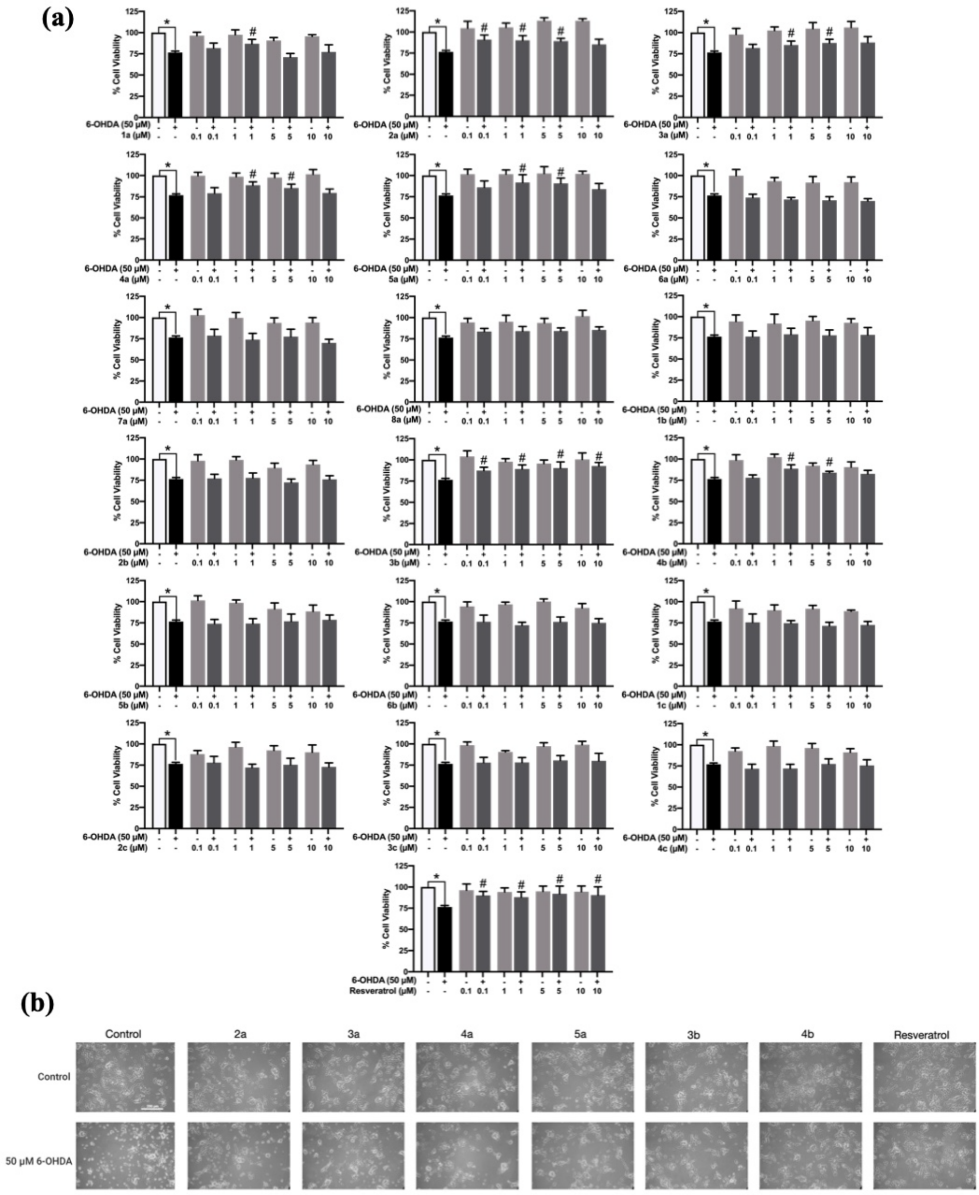
Neuroprotective activity of 1,2,3-triazole-based sulfonamide derivatives and resveratrol on 6-OHDA-induced SH-SY5Y neuronal cells. (a) Cell viability was assessed through the MTT assay (*N* = 5). Data are reported as the mean ± SEM and were statistically analyzed with one-way ANOVA. *p ≤ 0.05 compared to the control group. #*p* ≤ 0.05 compared to the 6-OHDA-induced group. (b) Observation of cell morphology of SH-SY5Y cells pretreated with 5 μM of selected 1,2,3-triazole-based sulfonamide derivatives (**2a** – **5a** and **3b** – **4b**) was made under an inverted light microscope with 40× magnification, scale bar = 200 μm.

### Effect of 1,2,3-Triazole-Based Sulfonamide Derivatives on ROS Production

ROS is essential for normal cellular functions such as cellular structure synthesis and host defense systems. However, in the presence of excessive production of ROS or impaired antioxidant defenses, the accumulated ROS could lead to oxidative stress, which is a condition that is harmful to several cellular structures (i.e., DNA, lipids, and proteins).^8^ The effect of 1,2,3-triazole-based sulfonamide derivatives on intracellular ROS production under 6-OHDA-induced stress was determined via the H_2_DCFDA assay. The results revealed that the cells induced by 50 μM 6-OHDA had a significant increase in intracellular ROS production (119.30 ± 4.745%) when compared to the control group (*p* ≤ 0.05). In contrast, pretreatment with all selected compounds (**2a** – **5a** and **3b** – **4b**) effectively decreased intracellular ROS production (101.6 ± 4.743%, 102.8 ± 4.944%, 103.0 ± 5.146%, 103.6 ± 4.425%, 102.9 ± 5.802%, and 102.5 ± 5.639%, respectively), similar to the resveratrol (101.1 ± 5.503%) when compared to the 6-OHDA-treated group (*p* ≤ 0.05) (Figure 4). These findings showed that the selected 1,2,3-triazole-based sulfonamide derivatives effectively reduced oxidative stress through the reduction of intracellular ROS accumulation in 6-OHDA-induced SH-SY5Y neuronal cells.

**Figure 4:**
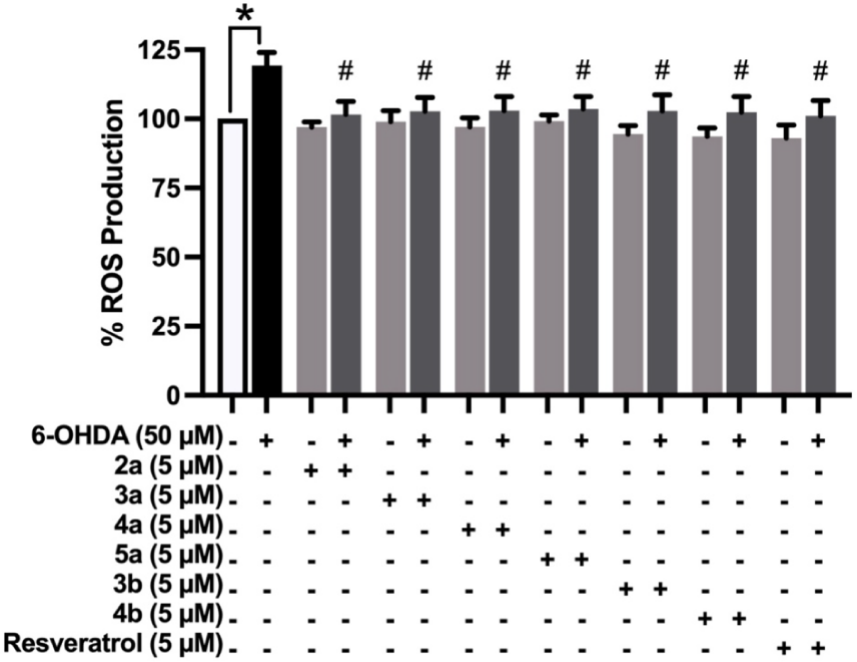
ROS production of 5 μM 1,2,3-triazole-based sulfonamide derivatives (**2a** – **5a** and **3b** – **4b**) and resveratrol on 6-OHDA-induced SH-SY5Y neuronal cells was determined by the H_2_DCFDA assay (*N* = 5). The data are shown as mean ± SEM. Statistical analysis was done with one-way ANOVA. *p ≤ 0.05 compared to the control group. #*p* ≤ 0.05 compared to the 6-OHDA-induced group.

### Effect of 1,2,3-Triazole-Based Sulfonamide Derivatives on Cellular Leakage of LDH

To evaluate the neuroprotective effect of the compounds, LDH release was measured. The SH-SY5Y cells were pretreated with 5 μM of 1,2,3-triazole-based sulfonamide derivatives (**2a** – **5a** and **3b** – **4b**) or resveratrol, followed by 6-OHDA as mentioned above, before the determination of LDH activity. The results revealed that LDH activity in the 6-OHDA-induced cells significantly increased (132.4 ± 5.089%) compared to the control groups (*p* ≤ 0.05), while the cells pretreated with the studied compounds showed significantly reduced LDH leakage (109.8 ± 3.826%, 112.6 ± 2.585%, 113.3 ± 6.585%, 116.0 ± 2.698%, 115.6 ± 2.926%, and 113.5 ± 2.954%, respectively) compared to the 6-OHDA-induced group (*p* ≤ 0.05). Pretreatment with resveratrol also showed significant improvement in LDH leakage (115.6 ± 2.723%) compared to the 6-OHDA-treated cells (*p* ≤ 0.05) (Figure 5). These findings suggested that the selected 1,2,3-triazole-based sulfonamide derivatives exhibited a protective effect against 6-OHDA-induced neurotoxicity through the inhibition of LDH release in SH-SY5Y cells.

**Figure 5:**
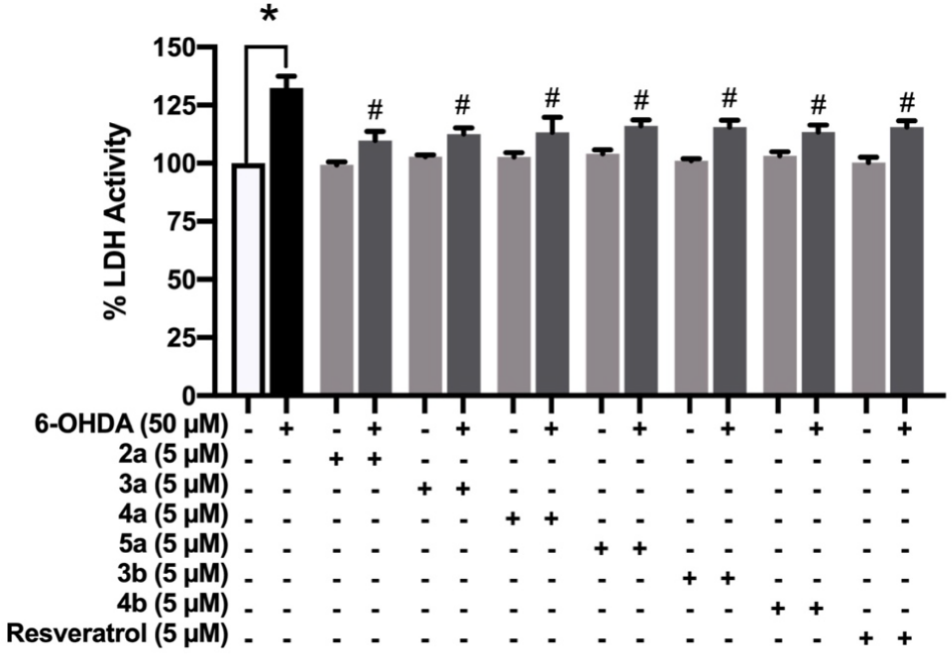
LDH released in culture medium of SH-SY5Y cells pretreated with 5 μM of 1,2,3-triazole-based sulfonamide derivatives (**2a**–**5a** and **3b** – **4b**) and resveratrol against 6-OHDA-induced SH-SY5Y neuronal cells (*N* = 5). Data are reported as mean ± SEM and evaluated statistically with one-way ANOVA. *p ≤ 0.05 compared to the control group. #*p* ≤ 0.05 compared to the 6-OHDA-induced group.

### Effect of 1,2,3-Triazole-Based Sulfonamide Derivatives on SIRT1 Deacetylase Activity

SIRT1 has been proven to play a critical role in modulating a wide range of physiological processes, including apoptosis, DNA repair, inflammatory response, metabolism, cancer, and stress. Reducing ROS and neuroinflammation by targeting SIRT1 may represent a promising therapeutic target for neurodegenerative disorders.^54,55^ Herein, the percentage of SIRT1 activity in the 6-OHDA-treated cells showed a significant decrease (78.78 ± 3.736%) compared to the control group. For 5 μM of 1,2,3-triazole-pretreated cells (**2a** – **5a** and **3b** – **4b**), the levels of SIRT1 activity were significantly increased (91.90 ± 1.452%, 92.53 ± 1.975%, 92.89 ± 3.187%, 93.74 ± 2.535%, 94.26 ± 3.277%, and 91.06 ± 0.5890%, respectively) compared to the 6-OHDA group (*p* ≤ 0.05). Resveratrol, the well-known SIRT1 regulator, also showed a significant increase in SIRT1 activity (92.61 ± 1.059%) compared to the 6-OHDA group (*p* ≤ 0.05) (Figure 6). Therefore, 1,2,3-triazole-based sulfonamide derivatives showed significant neuroprotective activity against 6-OHDA-induced neurotoxicity in SH-SY5Y cells, especially in activating SIRT1 protein.

**Figure 6:**
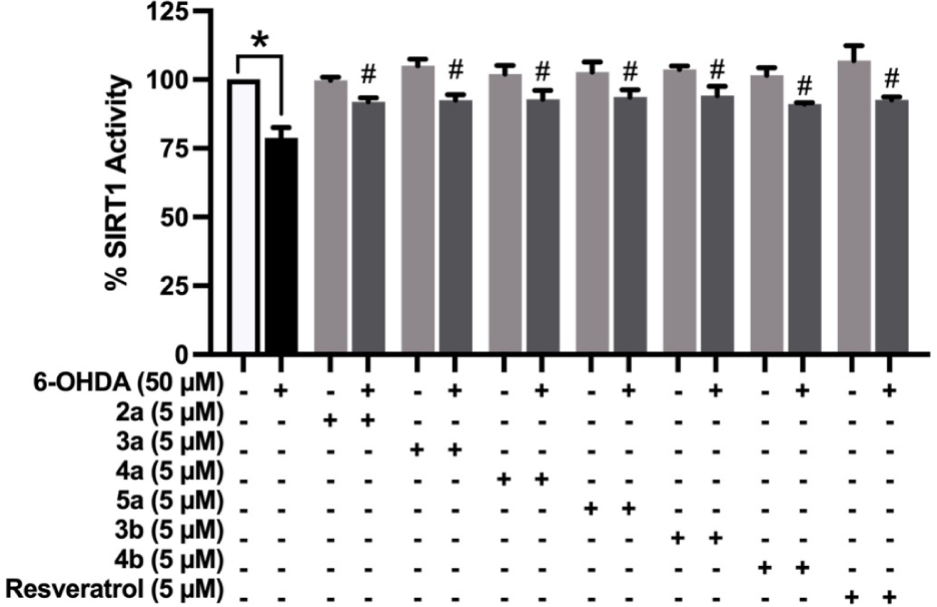
SIRT1 activity of 5 μM 1,2,3-triazole-based sulfonamide derivatives (**2a** – **5a** and **3b** – **4b**) and resveratrol pretreatment in SH-SY5Y cells against 6-OHDA-induced cell death (*N* = 5). Data are reported as mean ± SEM of percentages and statistically analyzed with one-way ANOVA. *p ≤ 0.05 compared to the control group. #*p* ≤ 0.05 compared to the 6-OHDA-induced group.

### *In Silico* Physicochemical and Pharmacokinetic Predictions of 1,2,3-Triazole-Based Sulfonamides

Lipinski’s rule of five is the first well-known rule for the determination of drug-likeness. According to Lipinski, a drug-like molecule is a small lipophilic compound (MW ≤ 500 g/mol and LogP ≤ 5) with molar reflectivity between 40 and 130, whose molecule contains no more than five and ten hydrogen bond donors and acceptors, respectively.^34^ Moreover, the Veber’s rule suggests that compounds with fewer rotatable bonds (≤10) and lower TPSA (≤140 Å^2^) have high oral bioavailability.^35^ Compounds that comply with these rules are considered to have high drug-likeness and high oral bioavailability. Herein, physicochemical properties of the selected compounds (**2a**, **4a**, **5a**, and **4b**) were predicted to assess their drugabilities according to Lipinski’s and Veber’s rules, as shown in Table 2. All compounds complied with the Lipinski’s rule of five, while compound **4a** violated the Veber’s rule due to its high TPSA. Pharmacokinetic profiles, such as absorption, distribution, metabolism, and excretion (ADME), were evaluated (Table 3). Most of the studied compounds possessed moderate water solubility (LogS = −5.27 to −4.60 Log mol/L) and high intestinal absorption (94.987–100%). However, some compounds (**3a**, **4a**, and **3b**) displayed low Caco2 permeability (LogPapp < 0.9 in 10^–6^ cm/s). The ability of the compounds to permeate the blood–brain barrier (BBB) into the central nervous system (CNS) is essential for the successful development of neuroprotective agents. Compounds **2a**, **3a**, and **5a** showed moderate BBB permeability (−1 ≤ LogBB ≤ 0.3), while compounds **4a**, **3b**, and **4b** showed low BBB permeability (LogBB < −1). Moreover, compounds **2a**, **4a**, **5a**, and **4b** revealed moderate CNS permeability (−3 ≤ LogPS ≤ −2), while compounds **3a** and **3b** displayed low CNS permeability (LogPS < −3). The predictions also indicated that all compounds can be metabolized by cytochrome P450 3A4 (CYP3A4) prior to being excreted by the kidney, with total clearance ranging from 0.694 to 0.859 Log mL/min/kg. Toxicity predictions showed that all compounds were categorized as class 3 toxicity (LD_50_ = 300 mg/kg). Most of the compounds (**2a** – **3a**, **5a**, **3b**, and **4b**) showed nonimmunogenic, carcinogenic, and mutagenic potentials. While compound **4a** was predicted to be nonimmunogenic, it possibly induced carcinogenicity and mutagenicity. Moreover, none of the compounds were predicted to display potential toxicity against targets in Tox-21 pathways, including nuclear receptor signaling pathways (i.e., aryl hydrocarbon receptor (AhR), androgen receptor (AR), androgen receptor ligand binding domain (AR-LBD), estrogen receptor alpha (ER), estrogen receptor ligand binding domain (ER-LBD), peroxisome proliferator-activated receptor gamma (PPAR-γ)), stress response pathways (i.e., nuclear factor (erythroid-derived 2)-like 2/antioxidant responsive element (Nrf2/ARE), heat shock factor response element (HSE), mitochondrial membrane potential (MMP), phosphoprotein (tumor suppressor) p53 (p53), and ATPase family AAA domain-containing protein 5 (ATAD5)).^56^

**Table 2:** Prediction of Physicochemical Properties of 1,2,3-Triazole-Based Sulfonamide Derivatives^a^

	Drug-likeness rules		Triazole-based derivatives					
	Lipinski’s	Veber’s	**2a**	**3a**	**4a**	**5a**	**3b**	**4b**
Formula	-	-	C_24_H_24_N_4_O_3_S	C_24_H_22_N_4_O_4_S	C_23_H_21_N_5_O_5_S	C_24_H_24_N_4_O_3_S	C_25_H_22_N_4_O_4_S	C_25_H_24_N_4_O_3_S
MW (g/mol)	≤500	-	448.54	462.52	479.51	448.54	474.53	460.55
Rotatable bond	-	≤10	9	10	10	9	7	6
H-bond acceptor	≤10	-	10	10	10	8	6	8
H-bond donor	≤5	-	1	1	1	1	0	0
Aromatic ring	-	-	3	3	3	3	3	3
Carbon atom	-	-	24	24	23	24	25	25
Heteroatom	-	-	8	9	11	8	9	8
LogP	≤5	-	3.66	3.03	2.58	3.65	2.99	3.61
TPSA (Å^2^)	-	≤140	94.49	111.56	140.31^b^	94.49	102.77	85.7

a MW, molecular weight; LogP, lipophilicity; TPSA, topological polar surface area.

b Violated Veber’s rule.

**Table 3:** Prediction of Pharmacokinetic Properties of 1,2,3-Triazole-Based Sulfonamide Derivatives^a^

	Interpretation		Triazole derivatives					
	High	Low	**2a**	**3a**	**4a**	**5a**	**3b**	**4b**
**Absorption**								
P-gp substrate	-	-	No	No	No	No	Yes	Yes
LogS (Log mol/L)	>0	<−10	–5.16	–4.6	–4.92	–5.16	–4.7	–5.27
LogPapp (in 10^‑6^ cm/s)	>0.9	<0.9	1.157	0.853	0.723	0.994	0.758	1.108
% Intestinal absorbance	>30	<30	95.379	97.783	96.2	94.987	100	99.094
**Distribution**								
LogBB	>0.3	<−1	–0.662	–0.872	–1.131	–0.66	–1.285	–1.056
LogPS	>−2	<−3	–2.465	–3.133	–2.746	–2.454	–3.006	–2.152
**Metabolism**								
CYP3A4 substrate	-	-	Yes	Yes	Yes	Yes	Yes	Yes
CYP2D6 substrate	-	-	No	No	No	No	No	No
**Excretion**								
Total clearance (Log mL/min/kg*)*	>0	≤−1	0.859	0.8	0.824	0.853	0.694	0.748
**Toxicity**								
Toxicity class	-	-	3	3	3	3	3	3
LD_50_ (mg/mL)	-	-	300	300	300	300	300	300
Carcinogenicity	-	-	I	I	A	I	I	I
Immunogenicity	*-*	*-*	_I_	_I_	_I_	_I_	_I_	_I_
Mutagenicity	-	-	I	I	A	I	I	I
Tox-21 signaling pathways	-	-	I	I	I	I	I	I

a LogS, water solubility (Log mol/L); LogPapp, Caco2 permeability (in 10^–6^ cm/s); LogBB, blood–brain barrier (BBB) permeability; LogPS, central nervous system (CNS) permeability; I, inactive, A, active.

### Molecular Docking of 1,2,3-Triazole-Based Sulfonamide Derivatives on SIRT1 Protein

Molecular docking was conducted to predict possible binding modes of the studied compounds against the binding site of the SIRT1 protein. The implementation of molecular docking could support advanced investigations into the potential interaction of 1,2,3-triazole-based sulfonamide derivatives with the SIRT1 protein. To provide reliability of the docking protocol, redocking of the cocrystallized resveratrol was performed, yielding an RMSD < 2.0 Å. Molecular docking was subsequently simulated on the selected triazole derivatives (**2a** – **5a** and **3b** – **4b**), and the models providing the highest occupying percentage were selected. The results showed that all compounds were well-occupied within the same binding site of the resveratrol on the SIRT1 protein (binding free energy: **2a** = −10.92, **3a =** −10.45, **4a** = −10.94, **5a** = −10.4, **3b =** −12.15, and **4b =** −11.77 kcal/mol, Figure 7a). These compounds showed comparable binding energy to that of resveratrol (−7.62 kcal/mol). 2D ligand-protein interaction diagrams revealed that PRO212, LEU215, and ILE223 are key amino acid residues that play critical roles in the binding of the 1,2,3-triazole-based sulfonamide derivatives against the SIRT1 target (Figure 7b).

**Figure 7:**
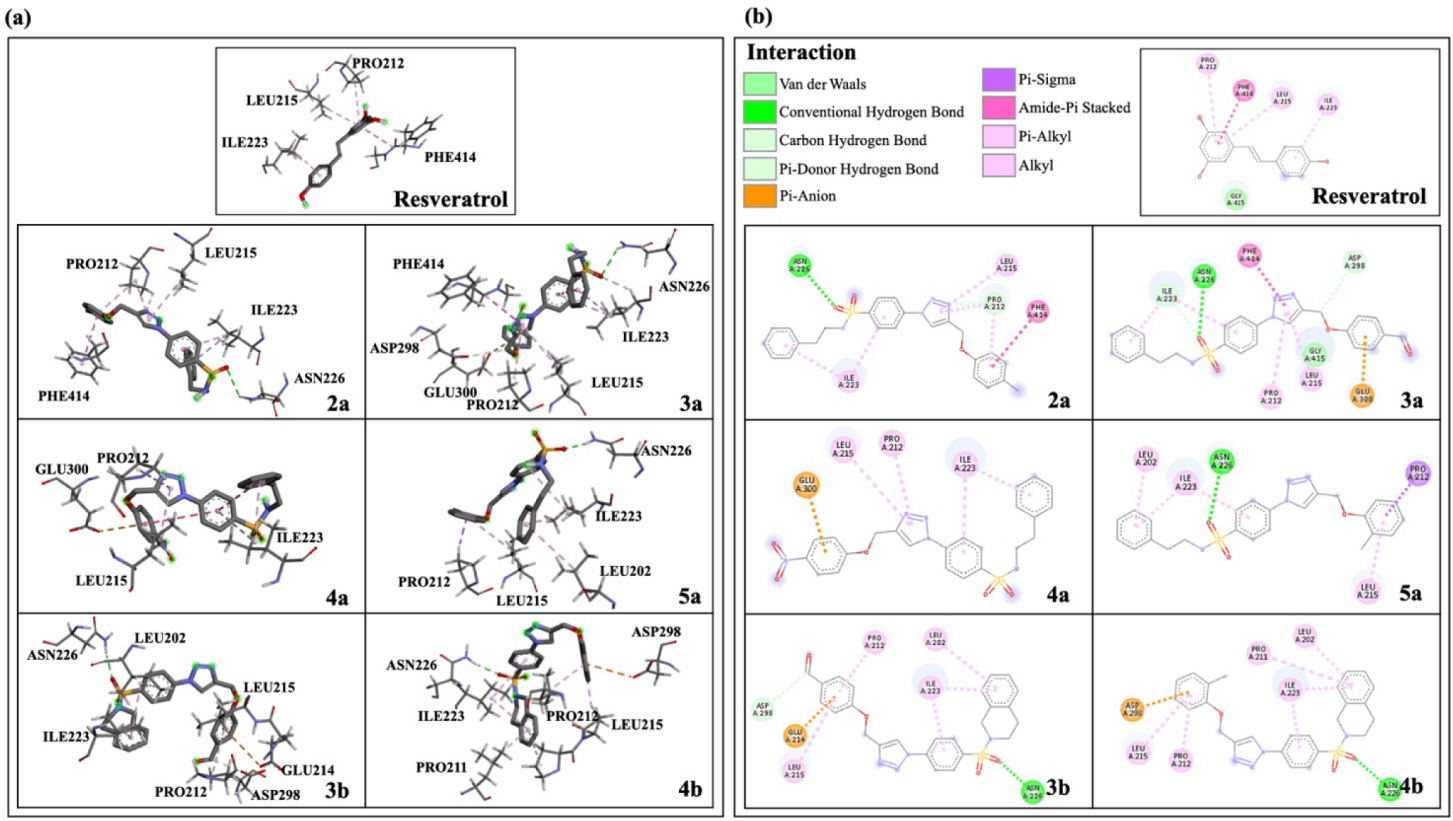
Molecular docking of selected 1,2,3-triazole-based sulfonamides (**2a** − **5a** and **3b** – **4b**) on the SIRT1 crystal structure (5BTR). (a) 3D docking conformations showing possible binding pose of each compound (**2a** – **5a**, **3b** – **4b**, and resveratrol) within the active binding site of SIRT1. (b) 2D illustration of molecular interactions between the triazole-based sulfonamide derivatives (**2a** – **5a** and **3b** – **4b**) and resveratrol. The key amino acids are shown in circles according to the type of interaction.

### Target Prediction of 1,2,3-Triazole-Based Sulfonamide Derivatives

Web-based tools were employed for predicting potential targets of the selected triazoles and PD-related targets. Results from three databases (i.e., SwissTargetPrediction, SEA, and SuperPred) revealed a data set of 361 unique potential targets of the compounds. A data set of 767 PD-related genes was acquired from three online databases (i.e., GeneCards, DisGeNET, and CTD). The shared common targets between the compounds and the disease data set were visualized through the Venn diagram, and it was identified that 54 targets from the compound data set were linked to PD (Figure 8a,b). Subsequently, these 54 common target proteins were integrated into the STRING database to construct the PPI network and visualized using Cytoscape 3.10.1 software. The proteins with higher degree values were illustrated in large red nodes, indicating their greater importance (Figure 8c). Eleven targets with higher degree centrality (DC) were listed as amyloid beta precursor protein (APP), nuclear factor kappa B subunit 1 (NFKB1), prostaglandin-endoperoxide synthase 2 (PTGS2), mechanistic target of rapamycin (MTOR), metabotropic glutamate receptor 5 (GRM5), nuclear factor erythroid 2-like 2 (NFE2L2), monoamine oxidase A (MAOA), sirtuin 1 (SIRT1), mitogen-activated protein kinase 8 (MAPK8), Src family tyrosine kinase Fyn (FYN), and mitogen-activated protein kinase 14 (MAPK14). These targets were considered core targets and were further analyzed for their key topological properties, including betweenness (BC) and closeness (CC) centralities (Table 4).

**Figure 8:**
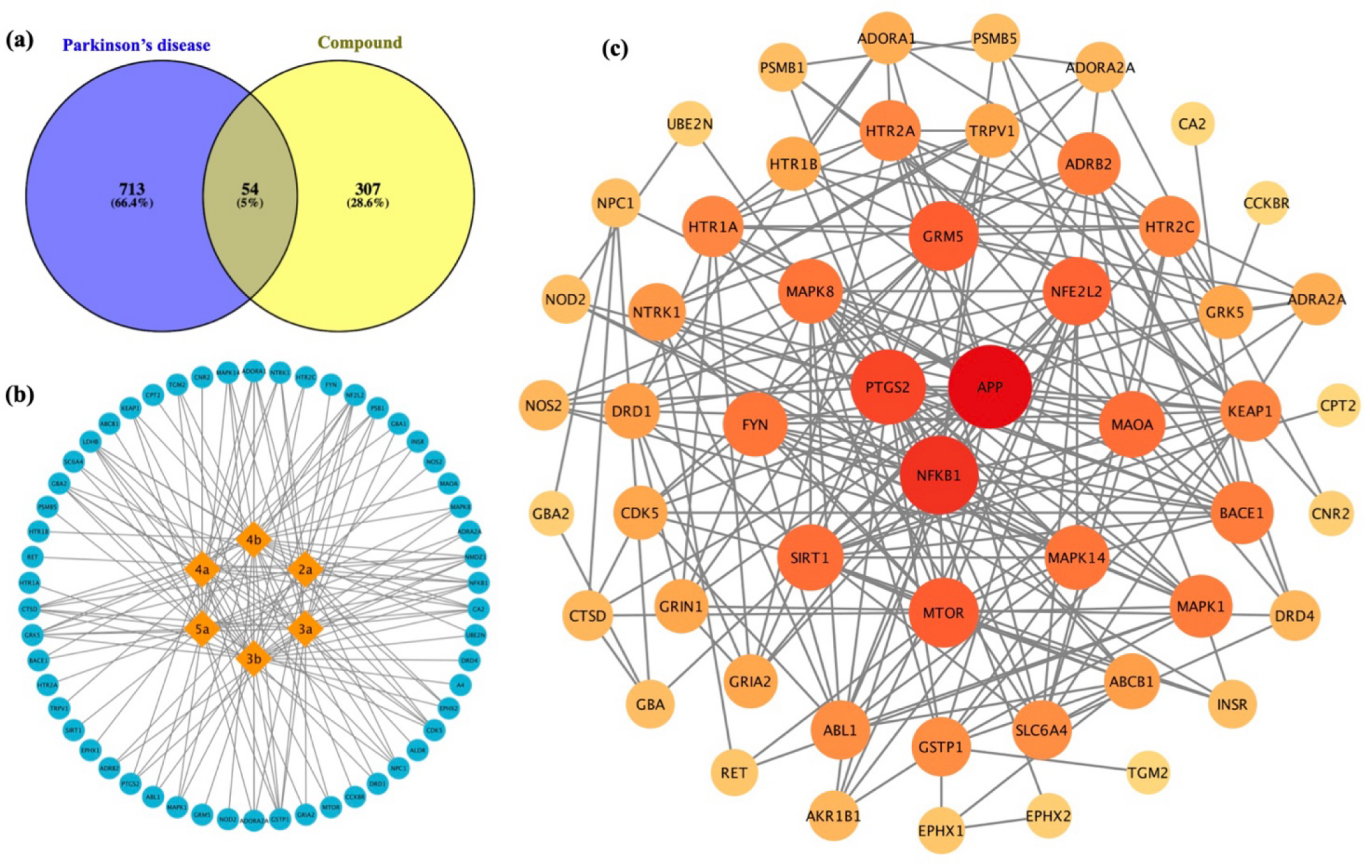
Illustration of the PPI network mapping. Common targets between the PD-related proteins and predicted protein targets of 1,2,3-triazole-based sulfonamides are represented in (a) Venn diagram and (b) compound-target network. 1,2,3-Triazole-based sulfonamide derivatives are represented as orange diamonds, while PD-related proteins are represented as blue circles. (c) PPI network diagram of the PD-related proteins (confidence level > 0.4) in the STRING database. PPI network is represented from a low to high degree with various colors, sizes, and layer forms. The large red circles in the innermost layer represent the highest degree, while the small yellow circles on the outermost layer represent the lowest degree.

**Table 4:** Core Target Information in the PPI Network of 1,2,3-Triazole-Based Sulfonamides^a^

No.	Target Name	Abbreviation	UniProtID	DC	BC	CC
1	Amyloid-β precursor protein	APP	P05067	24	0.268	0.627
2	Nuclear factor NF-kappa-B p105 subunit	NFKB1	P19838	21	0.118	0.565
3	Prostaglandin G/H synthase 2	PTGS2	P35354	19	0.127	0.598
4	Serine/threonine-protein kinase mTOR	MTOR	P42345	16	0.062	0.495
5	Metabotropic glutamate receptor 5	GRM5	P41594	16	0.042	0.525
6	Nuclear factor erythroid 2-related factor 2	NFE2L2	Q16236	15	0.036	0.520
7	Amine oxidase [flavin-containing] A	MAOA	P21397	14	0.080	0.525
8	NAD-dependent protein deacetylase sirtuin-1	SIRT1	Q96EB6	14	0.063	0.542
9	Mitogen-activated protein kinase 8	MAPK8	P45983	13	0.012	0.505
10	Tyrosine-protein kinase Fyn	FYN	P06241	13	0.035	0.525
11	Mitogen-activated protein kinase 14	MAPK14	Q16539	13	0.014	0.473

a DC, degree centrality; BC, betweenness centrality; CC, closeness centrality.

## Discussion

Degeneration of dopaminergic neurons is the main neuropathology of PD.^4^ Human neuroblastoma SH-SY5Y cell line is commonly used as an *in vitro* PD model due to its capability to differentiate into several types of neuronal phenotypes, including dopaminergic neurons.^57^ Hence, SH-SY5Y cells were selected for investigation in this study. To mimic the PD condition, 6-OHDA, a widely used dopamine analogue, was selected as an inducer capable of infiltrating into the cells via the dopamine transporter.^50^ Our studies demonstrated that after 24 h of exposure to various concentrations of 6-OHDA, neuronal cell viability was reduced in a dose-dependent manner. The neurotoxicity of 6-OHDA was found to be induced by several pathways, including oxidative stress and apoptosis.^50^ The results also revealed that 24 h of exposure to 6-OHDA significantly increased the intracellular ROS production of SH-SY5Y cells, as shown in the H_2_DCFDA assay. This could lead to oxidative stress and oxidative damage of cellular components (i.e., DNA damage, protein oxidation, and lipid peroxidation).^7^

In this study, a set of eighteen 1,2,3-triazole-based sulfonamides previously reported as anticancer agents by our group^25,26^ was investigated for their neuroprotective effects. These compounds exhibited low cytotoxicity against the normal embryonic lung MRC-5 cell line, suggesting that they are relatively safe. Moreover, some of these compounds were reported as aromatase inhibitors.^21^ Aromatase is an enzyme responsible for estrogen biosynthesis, which is mostly located in the brain.^58^ Though the relationship between neurodegeneration and aromatase is still unclear, previous reports revealed that elevated aromatase expression in the brain was found in several cases of brain injury and chronic neurodegeneration.^59^ Additionally, *in vivo* studies demonstrated the improvement of cognitive function after treatment with aromatase inhibitors.^59^ This suggests that compounds with aromatase inhibitory effects may potentially elicit neuroprotective activities.

Our study showed that the pretreatment of six 1,2,3-triazole-based sulfonamide derivatives (i.e., **2a** – **5a** and **3b** – **4b**) significantly restored the cell viability of the 6-OHDA-induced neuronal cells; thus, these compounds were selected for further investigations. The decrease in cell death, improvement in cell longevity, or increase in cell proliferation was found to be the major factors leading to the improvement in cell viability.^60^ Programmed cell death, including apoptosis and necrosis, is the process used to eliminate unwanted cells, such as damaged or infected cells.^61^ The process of necrosis or apoptosis would cause leakage of the cell membrane, leading to the release of cytoplasmic enzymes, such as LDH, into the extracellular medium. Hence, the increase in LDH activity in the cell culture medium could indicate the increase in cellular damage.^62^ In our study, pretreatment with selected compounds (i.e., **2a** – **5a** and **3b**– **4b**) effectively protects the cell membrane damage, as shown by significantly decreased levels of LDH leakage in the 6-OHDA-induced cells. Similar protection against LDH leakage and its relation to protection against cell death was also reported by our group for nitrogen-containing compounds.^29,63^

Considerable attention has been given to antioxidant compounds according to the role of oxidative stress in neurodegeneration.^8,64^ Clinical trials have highlighted various antioxidants, such as creatine, vitamin E, and coenzyme Q10, for their promising therapeutic effects against PD.^65^ It was revealed that the selected compounds (i.e., **2a** – **5a** and **3b** – **4b**) significantly reduce the levels of intracellular ROS in 6-OHDA-induced cells, suggesting their antioxidative properties. However, the decrease in ROS levels could possibly be due to their abilities in inhibiting the production of ROS or enhancing the activity of antioxidant enzymes (i.e., superoxide dismutase (SOD) and catalase (CAT)).^66^ In our previous study, the neuroprotective effects of nitrogen-containing heterocyclic compounds were found to be due to their antioxidative properties, which enable them to regulate SOD2, upregulate antiapoptotic (BCL-2), and downregulate the apoptotic (BAX) proteins.^67^ Additionally, our group reported that triazole derivatives exhibit antiapoptotic and mitochondrial protective properties via regulating SOD2 along with modulating the SIRT1/2/3-FOXO3a pathway.^68^

SIRT1 is a crucial survival protein eliciting cellular protective effects via various mechanisms, including oxidative stress.^69^ SIRT1 mitigates oxidative stress-induced damage and regulates key antioxidant enzymes (i.e., SOD and CAT).^70^ Moreover, SIRT1 is the upstream protein of the forkhead box O (FoxO) protein.^71^ An activation of SIRT1 protein has been reported to suppress the expression of the FoxO3a gene, leading to a decrease in oxidative stress.^72^ SIRT1 regulates several cellular functions (i.e., DNA transcription, cell cycle progression, and inflammation), which are crucial for cellular survival.^73^ SIRT1 also plays an important role in the antiapoptotic effect through the deacetylation of the p53 protein, a tumor suppressor gene highly expressed in damaged neurons.^74,75^ Additionally, SIRT1 regulates neuroinflammation via suppressing the regulatory inflammatory cytokines (i.e., tumor necrosis factor-α (TNF-α), interleukin-1β (IL-1β), and interleukin-6 (IL-6)) in microglial cells.^76^ SIRT1 also regulates the degradation of α-synuclein oligomers via upregulating the heat shock protein 70.^77^ Resveratrol is a neuroprotective agent, which is well-known as a SIRT1 activator.^78^ Our findings suggested that the 1,2,3-triazole-based sulfonamide derivatives (i.e., **2a** – **5a** and **3b** – **4b**) enhance SIRT1 protein activity, with comparable potency to that of the resveratrol. This was supported by the molecular docking study, which indicated that these triazoles could occupy the same binding site as resveratrol on the target SIRT1 protein. These compounds shared common binding amino acid residues with that of the resveratrol. However, an additional hydrogen bond formation, which is absent in the binding of resveratrol, was observed between the sulfonamide group and ASN226 for all compounds, except for **4a**. Triazole ring was revealed to play a part in pi-alkyl interaction with LEU215 and PRO212 residues, as observed for **2a** – **4a** (Figure 7). SIRT1 is a promising therapeutic target for PD models. Several compounds, such as atractylenolide-I (ATR-I),^79^ baicalein,^80^ and urolithin A,^81^ showed promising neuroprotective activities against PD model through oxidative stress modulation, improvement of mitochondrial function, and upregulation of SIRT1 activity and its related pathways such as PGC-1α, Nrf2, AMPK, and mTOR. In comparison, selected 1,2,3-triazole-based sulfonamide derivatives (i.e., **2a** − **5a** and **3b** – **4b**) also possess significant activation of the SIRT1 protein while reducing oxidative stress in the 6-OHDA-induced SH-SY5Y neurotoxicity. Studies have also shown the neuroprotective properties of other 1,2,3-triazole-based sulfonamide derivatives in oxidative stress-induced SH-SY5Y cell line through the activation of the SIRT1-FOXO3a signaling pathway.^68^ These findings suggest that the selected 1,2,3-triazole-based sulfonamide derivatives (**2a** – **5a** and **3b** – **4b**) could serve as promising SIRT1-targeting neuroprotective agents.

Target prediction revealed that there are 54 common shared targets between the studied 1,2,3-triazole-based sulfonamide derivatives and PD-related targets. Among these, 11 core targets with high degree centrality were highlighted as core targets with high influence and could play important roles in several PD-related pathways, such as oxidative stress, inflammatory pathways, cellular senescence pathways, and neurotransmitter regulation. In response to oxidative stress, several studies target the Nrf2 signaling pathway for potential antioxidative strategies for PD.^82^ Neuroinflammation is found to be one of the key factors leading to PD pathogenesis,^83^ which suggests the suppression of neuroinflammation as one of the therapeutic approaches.^84,85^ Our predictions suggested several inflammatory-related targets as potential targets for the investigated triazoles such as NFKB1, PTGS2 (or COX-2), MAPK8 (or JNK1), and MAPK14 (or p38). These proteins were found to be highly involved in neuroinflammation in PD. For instance, the presence of α-synuclein aggregation could lead to the translocation of NF-κB, a protein transcription factor that is responsible for innate immunity regulation, in microglial cells.^84^ This results in an increase in proinflammatory cytokine release and subsequently causes mitochondrial damage and degeneration of dopaminergic neurons.^84^ Moreover, our 1,2,3-triazole-based sulfonamide derivatives were predicted to modulate the metabolism of dopamine via modulating the MAO, an enzyme involved in dopamine breakdown.^86^ The combined use of MAO inhibitors together with levodopa is noted as one of the effective strategies for PD treatment.^14^ Additionally, mTOR, a key regulatory protein involved in various cellular processes (i.e., cell growth, proliferation, and survival), is also predicted as one of the potential core targets. Dysregulation of mTOR signaling is implicated in various neurodegenerative diseases, including PD,^87^ and plays roles in abnormal protein aggregation (i.e., α-synuclein, autophagy, and mitochondrial function).^88,89^

*In silico* pharmacokinetic prediction has drawn attention in increasing the success rate and decreasing late-stage failures in drug development.^90^ Our predictions suggested that the selected compounds (**2a**, **3a**, **5a**, **3b**, and **4b**) are drug-like, possessing preferable intestinal absorption (>90%), which indicates their possibilities to be further developed as convenient oral drugs.^91^ However, some compounds are unable to pass across the BBB (**1a** – **3a** and **5a**) or CNS (**1a** – **2a** and **4a** – **5a**), which limits their access to the target site of action in the CNS.^92^ However, this limitation can be possibly overcome by structural optimization and a drug delivery system to enhance distribution to the target site of action. Additionally, alternative routes of administration (i.e., intranasal and intrathecal routes) can also bypass the BBB as well as enhance bioavailability.^92^ Combined therapy may also be useful to address this issue.^93^ In addition to poor pharmacokinetic profiles, toxicity is another factor leading to failure in drug development.^94^ Findings from the toxicity prediction suggest that most of the investigated triazoles might be further developed as relatively safe therapeutic agents.

## Conclusion

A series of 1,2,3-triazole-based sulfonamides was synthesized and evaluated for their neuroprotective activities against 6-OHDA-induced neuronal damage in SH-SY5Y cells. Six compounds from type I and type II (**2a** – **5a** and **3b** – **4b**) showed low cytotoxicity toward normal MRC-5 cells and demonstrated neuroprotective effects against SH-SY5Y cells by improving cell viability, decreasing ROS production, and reducing LDH leakage. Moreover, the compounds enhanced the SIRT1 deacetylase activity (Figure 9). This was supported by a molecular docking study, which revealed possible binding modes and potential interactions of these derivatives within the active binding site of SIRT1. Sulfonamide and triazole moieties were highlighted as key interaction features against the ILE223, LEU215, and PRO212 residues. Target prediction revealed a set of 11 potential core targets associated with oxidative stress, inflammatory pathways, cellular senescence pathways, and neurotransmitter regulation. Pharmacokinetic predictions also indicated that the selected triazoles complied with the Lipinski’s rule of five and Veber’s rule, suggesting their preferable drug-likeness. Overall, the synthesized (**2a** – **5a** and **3b** – **4b**) derivatives possess promising neuroprotective properties, making them potential candidates for further development as PD therapeutic agents. However, further *in vivo* studies and clinical investigations are recommended to validate their efficacy and safety in a broader context.

**Figure 9:**
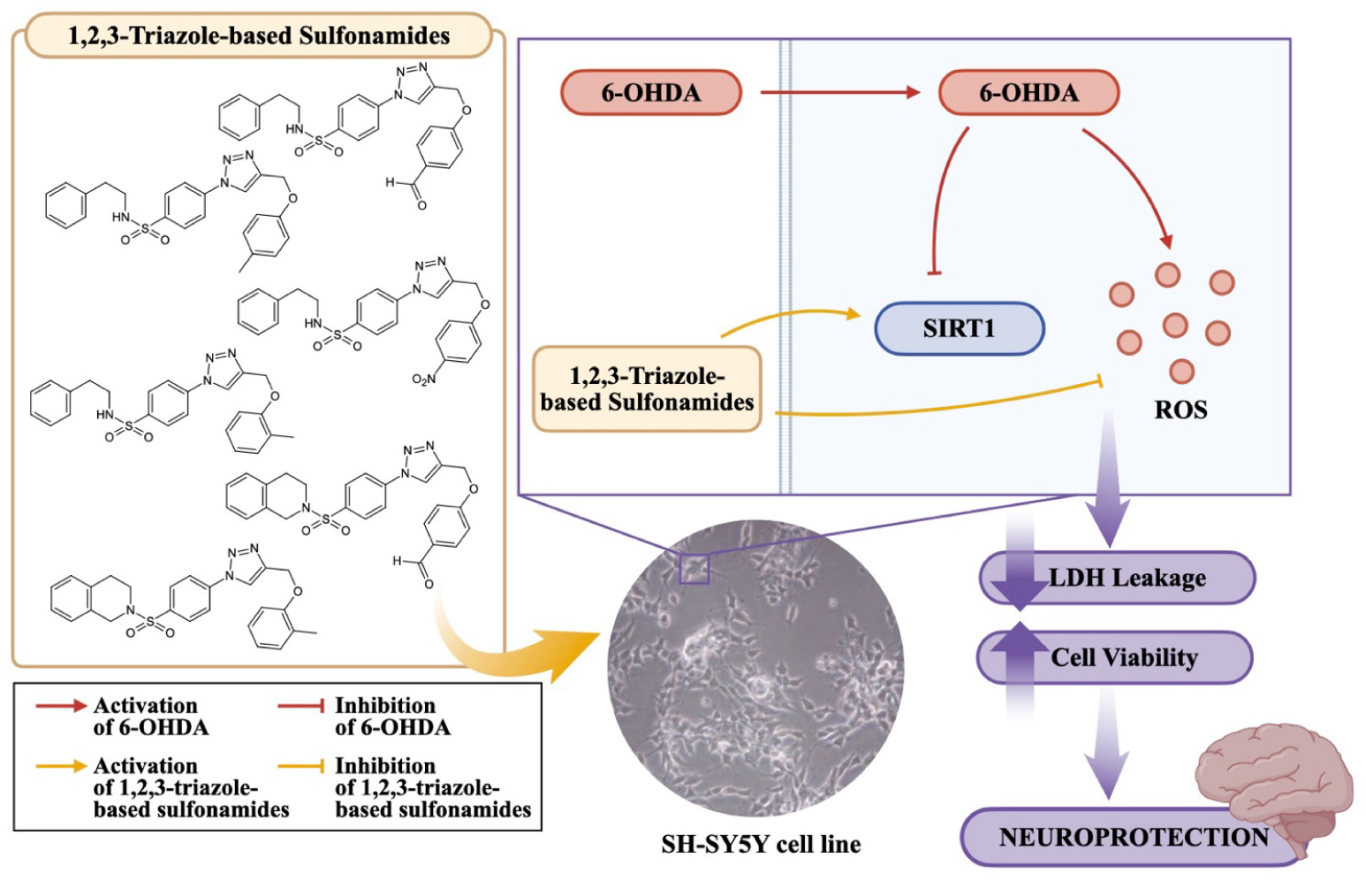
Summary of neuroprotective activity of 1,2,3-triazole-based sulfonamides by improving cell viability, decreasing ROS production, and reducing LDH leakage through the activation of the SIRT1 protein.
